# Abdominal Tumor Characterization and Recognition Using Superior-Order Cooccurrence Matrices, Based on Ultrasound Images

**DOI:** 10.1155/2012/348135

**Published:** 2012-01-19

**Authors:** Delia Mitrea, Paulina Mitrea, Sergiu Nedevschi, Radu Badea, Monica Lupsor, Mihai Socaciu, Adela Golea, Claudia Hagiu, Lidia Ciobanu

**Affiliations:** ^1^Department of Computer Science, Technical University of Cluj-Napoca, George Baritiu Street 26–28, 400027 Cluj-Napoca, Romania; ^2^Department of Ultrasonography, Iuliu Hatieganu University of Medicine and Pharmacy, Victor Babeş Street 8, 400079 Cluj-Napoca, Romania

## Abstract

The noninvasive diagnosis of the malignant tumors is an important issue in research nowadays. Our purpose is to elaborate computerized, texture-based methods for performing computer-aided characterization and automatic diagnosis of these tumors, using only the information from ultrasound images. In this paper, we considered some of the most frequent abdominal malignant tumors: the hepatocellular carcinoma and the colonic tumors. We compared these structures with the benign tumors and with other visually similar diseases. Besides the textural features that proved in our previous research to be useful in the characterization and recognition of the malignant tumors, we improved our method by using the grey level cooccurrence matrix and the edge orientation cooccurrence matrix of superior order. As resulted from our experiments, the new textural features increased the malignant tumor classification performance, also revealing visual and physical properties of these structures that emphasized the complex, chaotic structure of the corresponding tissue.

## 1. Introduction

The hepatocellular carcinoma (HCC) is the most frequent malignant liver tumor, representing 75% of the liver cancer cases [1]. The colorectal tumors also represent a frequent disease for the population of the developed countries. The human observations are not enough in order to perform the detection of the malignant tumors, the resulted diagnosis accuracy being below 80%. The golden standard for cancer diagnosis is the biopsy, but this is an invasive, dangerous method that can lead to the spread of the tumor inside the human body. A non-invasive, subtle analysis is due, in order to detect the cancer in early evolution stages, when the tumor can be surgically removed. We perform this study by using computerized methods applied on ultrasound images. Other types of image acquisition techniques, such as computer tomography (CT), magnetic resonance imaging (MRI), and endoscopy are considered invasive or expensive. The texture is an important feature, as it provides subtle information concerning the pathological state of the tissue, overcoming the accuracy of the human perception, through the statistical and multiresolution approaches. The texture-based methods in combination with classifiers were widely used in the domain of malignant tumor characterization and recognition from medical images. In [2], Raeth used the textural features in order to distinguish the normal liver from the diffuse liver diseases and from the malignant liver tumors. The features derived from the second-order grey levels cooccurrence matrix, from the edge cooccurrence matrix, as well as other edge and gradient-based features, speckle noise distribution parameters, and the Fourier power spectrum, provided satisfying results concerning the differentiation between the tumoral and nontumoral tissue. In [3] the authors computed the first-order statistics (the mean grey level and the grey level variance), the second-order grey level cooccurrence matrix parameters and run-length matrix parameters which were used in combination with an artificial neural networks based classifier, as well as with a classifier based on linear discriminants in order to differentiate the malignant liver tumors from hemangioma and from the normal liver. The resulted recognition rate was 79.6%. The wavelet transform was also implemented [4], in order to perform a multi-resolution analysis of the textural features. The method provided satisfying results concerning the differentiation between malignant and benign liver lesions, the area under the ROC (receiver operating characteristic) curve being approximately 90%. In [5] the authors analyzed the fluorescent images of the colonic tissue based on textural parameters derived from the second order grey level cooccurrence matrix (GLCM), in order to distinguish the colonic healthy mucosa versus adenocarcinoma. However, a systematic study concerning the most relevant textural features that best characterize the malignant tumors and of the most appropriate methods that lead to an increased diagnosis accuracy is not done. We perform this in our work by building the imagistic textural model of the malignant tumors. We previously defined the imagistic textural model of the malignant tumors [6], consisting in the most relevant textural features able to separate the HCC tumor from the visually similar tissues (cirrhotic parenchyma, benign tumors), together with their specific values (mean, standard deviation, and probability distribution). In this work, we analyzed new methods for textural features computation, based on the superior order grey level cooccurrence matrix (GLCM) [7], respectively on the superior order edge orientation cooccurrence matrix (EOCM), the purpose being to improve the characterization of the abdominal malignant tumors, and to increase the automatic diagnosis accuracy. In this way, we expect to get a more subtle evaluation procedure than in the case of using the other textural features. The superior order GLCM was theoretically described by Akono in [7]. The third-order GLCM was experimented for the analysis of the trabecular bones in proximal femur radiographs [8], as well as for crop classification [9], but it was never implemented for tumor characterization and recognition. There are no important realizations in the image analysis domain involving the fifth-order GLCM matrix. The second order EOCM was implemented by Raeth in [2] for malignant tumor contour characterization and provided satisfying results in this domain. The third order EOCM was not previously implemented. Thus, we analyzed the role that the second-, third-, and fifth-order GLCM, respectively, the second- and third-order EOCM have, concerning both the subtle characterization of HCC and colonic tumor tissue, as well as the automatic diagnosis of these types of cancer. Extended Haralick features were defined for the characterization of the tumor texture, and the best orientations of the corresponding displacement vectors were determined in both cases of the superior order GLCM and EOCM. The edge orientation variability feature was also defined in order to characterize the complex structure of the tumor tissue. The malignant tumors were compared with visually similar tissues. The HCC tumor was compared with the cirrhotic liver parenchyma on which it had evolved and with the benign liver tumors. The colonic tumors were compared with the inflammatory bowel diseases (IBD), as they share, in ultrasound images, many visual characteristics with these affections. The assessment of the relevant textural features for the characterization of the malignant tumors was also performed, through specific methods such as the correlation-based feature selection (CFS) [10] and through the evaluation of the individual attributes based on their information gain with respect to the class [10]. Powerful classifiers that gave the best results in our former experiments [6], such as the multilayer perceptron [11] and the support vector machines (SVM) [11], as well as the AdaBoost combination scheme [11], were adopted for the evaluation of the textural model and of the recognition accuracy. The correlation of the textural features with the internal structure and with the properties of the tumor tissue was also discussed.

## 2. Materials and Methods

### 2.1. Materials and Working Methodology

In our study, mainly the patients suffering from HCC and colonic tumors were taken into consideration. Patients affected by benign liver tumors such as hemangioma and focal nodular hyperplasia (FNH) were also considered, being known that these tumors have a similar visual aspect with HCC in many situations. Subjects suffering from inflammatory bowel diseases (IBD) were taken into account as well, because these affections provided a similar visual aspect of the bowel walls like those provided by the colorectal tumors. All these patients were previously biopsied. For each patient, multiple images were acquired, corresponding to various orientations of the transducer, using the same settings of the ultrasound machine. The same number of images was considered for each patient, as described in the experimental section. Thus, the study was independent from the patient's characteristics. B-mode ultrasonography was used, in order to preserve the textural properties of the tissues. Rectangular regions of interest were selected inside the tumors, on the liver tissue, or on the bowel wall, in areas which were not affected by artifacts. Then, the imagistic textural model of the malignant tumors was built according to the steps below, and the role of the new derived textural features in improving the accuracy of the malignant tumor characterization and recognition performance was analyzed.

### 2.2. The Imagistic Textural Model of the Malignant Tumors

#### 2.2.1. The Imagistic Textural Model of the Malignant Tumors and the Phases Due for Model Building

The imagistic textural model of HCC consists of the set of relevant, independent textural features, able to distinguish this tumor from the cirrhotic liver parenchyma and from the benign tumors. The specific, statistical values of the textural features—mean, standard deviation, and probability distribution—are part of the model. The mathematical description of the imagistic textural model is given below. Let *F* be the space of the potentially relevant textural features, containing a number of *n* such features:

(1) $$F={\left\{f_{i}\right\}}_{i=1,\ldots ,n}.$$

The features from *F* are considered in their initial representation, as they appear after applying the image analysis methods. We define

(2) $$F_{R}=\text{Dimensionality\_reduction}\left(F\right)$$

as being the transformed feature space, obtained from the initial feature space, *F*, after applying dimensionality reduction methods—mainly feature selection techniques [10]. The imagistic textural model of the tumor (TM) consists of a collection of vectors *V*_*f*_*r*__, associated with each relevant textural feature *f*_*r*_, containing the specific values that characterize each analyzed class:

(3) $$\begin{array}{cc} \text{TM} & =\{V_{f_{r}}\;|\;V_{f_{r}}\\ & \;\;=[\text{Relevance},\text{Mean},\text{Standard}\_\text{deviation},\\ & \;\;\;\;\;\;\text{Probability}\_\text{distribution}]\}. \end{array}$$

The vectors of the imagistic textural model are composed by the specific parameters described by (3), where mean (the arithmetic mean value) and standard deviation are real numbers; the Relevance, represented by an integer, quantifies the importance that the considered textural feature has in the differentiation between HCC and other kinds of tissues.

In order to generate a reliable imagistic textural model, first, the image selection for the training set building is due. For each considered type of tissue, a corresponding class is built. Then, an image analysis phase is necessary: the textural feature computation using specific methods for texture analysis is involved in this process. The values of the textural features are stored in the database and used for further evaluations. The learning phase is essential in order to perform the relevant feature selection, to eliminate the redundant features and to determine the specific, statistical values, and the corresponding probability distributions. Dimensionality reduction methods consisting of feature selection [10] and feature extraction techniques [11] are implemented in this phase. At the end, a validation phase is necessary, involving the evaluation of the generated model by providing the relevant features at the classifiers inputs and estimating the accuracy of each classifier. A new test set of images, different from the training set, is used in this phase. The phases due in order to build the imagistic textural model are described below.

#### 2.2.2. Training Set Building

For each patient, three to five images were considered. On each image, rectangular regions of interest were selected on each type of tissue, inside HCC and the colonic tumors, respectively, on the cirrhotic parenchyma on which HCC evolved, as well as inside the benign liver tumors and on the superior part of the bowel wall affected by inflammatory bowel diseases. Pairs of classes were considered, and then the classes were combined in equal proportions inside the training set. The potentially relevant textural features were determined on the regions of interest, using specific methods for texture analysis, and the corresponding values were stored. An instance of the training set consisted of the values of the considered textural features, computed inside a certain region of interest, followed by the class specification.

#### 2.2.3. Methods Applied during the Image Analysis Phase

During the image analysis phase, noise reduction was initially performed, by using an averaging filter [12]. Then, specific methods for texture analysis were applied, providing the initial set of potentially relevant textural features. We previously computed 48 textural features, from the following categories: the mean value of the grey levels [12], the second order grey levels cooccurrence matrix (GLCM), and the associated Haralick parameters [13]—the energy, entropy, correlation, contrast, variance, and local homogeneity that emphasized the global properties of the texture. Edge and gradient-based statistics [12], respectively, the frequency and density of the textural microstructures, detected by using the Laws convolution filters were computed as well [12]. The Shannon entropy [14], computed after applying the wavelet transform [15], was also determined. The Haar wavelet transform was applied recursively at two levels of resolution: the low-low, low-high, high-low, and high-high components were derived at the first level, then, the wavelet transform was applied again on each of these components. The Shannon entropy was computed on each resulted component, at both first and second levels. The determined textural features were independent on orientation, as they were computed on multiple directions and the result was averaged. They were also independent of illumination and scaled with the size of the region of interest. In this work, we defined and experimented the third-and fifth order GLCM, respectively, the second-and third order EOCM, for obtaining more refined textural features. The effect of the new textural features on the improvement of the imagistic textural model of the malignant tumors was carefully analyzed.

#### 2.2.4. Description of the Learning Phase

During the learning phase, the selection of the relevant textural features was performed. We considered a feature as being relevant if it emphasized the defining characteristics of the tumor tissue and it substantially contributed to the separation of the tumor tissue from the visually similar tissues. From a more technical point of view, a feature was considered relevant if, by including it in the feature set, it led to an increase in the classification accuracy. There are specific methods for feature selection, integrated in two main groups, filters and wrappers [10], which perform a reliable separation of the relevant features from the nonrelevant ones. We compared, in our previous research [6], various methods from these categories, as well as their combinations. The best results were obtained when using the methods of correlation-based feature selection (CFS), combined with genetic search [10], the information gain attribute evaluation [16], the consistency-based feature subset evaluation [10], respectively the wrapper that used the decision trees as classifier, and the best first search method [16] for subset finding. The specific values of the relevant textural features were determined by using confidence intervals and probability distribution tables [11]. In this work, we assessed the relevance of the newly obtained textural features, by using the most powerful feature selection methods, being interested in the diagnosis accuracy improvement.

#### 2.2.5. Description of the Validation Phase

The validation phase consisted of providing the final set of relevant textural features at the inputs of some powerful classifiers, and in analyzing their effect on the classification process improvement. Classifiers from different categories, as well as classifier combinations, were compared in order to obtain the best performance during this phase [6]. The best results were provided by the methods of support vector machines (SVM) [11] with polynomial kernel of 3rd degree, by the multilayer perceptron (MLP), decision trees (C4.5 method), respectively, by the AdaBoost combination scheme. The following parameters were used in order to assess the classification performance: the recognition rate (percent of correctly classified instances), the sensitivity (TP rate), the specificity (TN rate), the area under the ROC curve (AUC) [11], and the time due for model building [16]. The stratified cross-validation strategy [11] was implemented for classification performance evaluation, in order to preserve the original class proportions.

### 2.3. The Newly Defined Textural Features and Their Role in the Improvement of Imagistic Textural Model

#### 2.3.1. The Description of the New Texture Analysis Methods

**(1) The Grey Level Cooccurrence Matrix of Superior Order**

The grey level cooccurrence matrix (GLCM), also called the Grey Tone Difference Matrix, was previously defined by Julesz et al. [17] and Haralick [18]. Julesz et al. [17] was the first who used grey tone spatial dependence cooccurrence statistics in his texture discrimination experiments. Haralick [18] defined the two-dimensional cooccurrence matrix of the grey levels as containing, in its elements, the number of pairs of pixels having two specific values of the intensity, *g*_1_ and *g*_2_, being situated at a distance defined by a displacement vector:

(4) $$\vec{d}=\left(\vec{d}x,\vec{d}y\right).$$

Haralick also defined and implemented statistical measures, such as the homogeneity, energy, entropy, correlation, variance, contrast [18], in order to emphasize the global properties of the texture. In [7], Akono et al. described the GLCM of order *n* and proposed a fast computation algorithm for this method, but did not state a corresponding definition. He also extended the mathematical expressions of several statistical (Haralick) measures from order two to order *n*, such as the sum of the GLCM elements, the inverse difference, the dissimilarity and the contrast.

We defined the GLCM of order *n* in the following manner:

(5) $$\begin{array}{cc} & C_{D}\left(g_{1},g_{2},g_{3},\ldots ,g_{n}\right)\\ & \;=\#\{\left(\left(x_{1},y_{1}\right),\left(x_{2},y_{2}\right),\left(x_{3},y_{3}\right),\ldots ,\left(x_{n},y_{n}\right)\right):\\ & \;\;\;\;f\left(x_{1},y_{1}\right)=g_{1},f\left(x_{2},y_{2}\right)=g_{2},\ldots ,\\ & \;\;\;\;f\left(x_{n},y_{n}\right)=g_{n},\left|x_{2}-x_{1}\right|=\left|\vec{d}x_{1}\right|,\\ & \;\;\;\;\left|x_{3}-x_{1}\right|=\left|\vec{d}x_{2}\right|,\ldots ,\left|x_{n}-x_{1}\right|=\left|\vec{d}x_{n-1}\right|,\\ & \;\;\;\;\left|y_{2}-y_{1}\right|=\left|\vec{d}y_{1}\right|,\left|y_{3}-y_{1}\right|=\left|\vec{d}y_{2}\right|,\ldots ,\\ & \;\;\;\;\left|y_{n}-y_{1}\right|=\left|\vec{d}y_{n-1}\right|,\;\\ & \;\;\;\;\mathrm{sgn}\left(\left(x_{2}-x_{1}\right)\left(y_{2}-y_{1}\right)\right)=\mathrm{sgn}\left(\vec{d}x_{1}\cdot \vec{d}y_{1}\right),\ldots ,\\ & \;\;\;\;\;\mathrm{sgn}\left(\left(x_{n}-x_{1}\right)\left(y_{n}-y_{1}\right)\right)=\mathrm{sgn}\left(\vec{d}x_{n-1}\cdot \vec{d}y_{n-1}\right)\}. \end{array}$$

In (5), #*S* is the number of the elements in the set *S*, while

(6) $$\vec{d}=\left(\left(\vec{d}x_{1},\vec{d}y_{1}\right),\left(\vec{d}x_{2},\vec{d}y_{2}\right),\ldots ,\left(\vec{d}x_{n-1},\vec{d}y_{n-1}\right)\right)$$

is the set of the displacement vectors. Thus, the GLCM matrix of order *n* contains in its elements the number of *n*-tuples of pixels with the spatial coordinates (*x*_*i*_, *y*_*i*_), *i* ∈ {1,…, *n*}, having the intensity values *g*_*i*_, *i* ∈ {1,…, *n*}, and being in a spatial relation defined by the displacement vectors described in (6). In practice, we used the GLCM probability matrix:

(7) $$p\left(g_{1},g_{2},\ldots ,g_{n}\right)=\frac{C_{D}\left(g_{1},g_{2},\ldots ,g_{n}\right)}{\sum_{g_{1}=0}^{N_{g}-1}\sum_{g_{2}=0}^{N_{g}-1}\ldots \sum_{g_{n}=0}^{N_{g}-1}C_{D}\left(g_{1},g_{2},\ldots ,g_{n}\right)}.$$

In (7), *N*_*g*_ is the total number of the gray levels in the image. Based on the *n*th order GLCM, we computed the following parameters: energy, entropy, local homogeneity, correlation, contrast, variance, as described in Appendix B. The maximum probability for a certain combination of grey levels to appear within the texture is also computed, as indicated in Appendix B, while searching for a specific pattern of grey levels within each type of analyzed tissue. The second order GLCM was determined for the following directions of the displacement vectors: 0°, 45°, 90°, and 135°. The corresponding Haralick features were averaged for all the resulted matrices.

**The Implementation of the Third Order GLCM**

**Figure 1:**
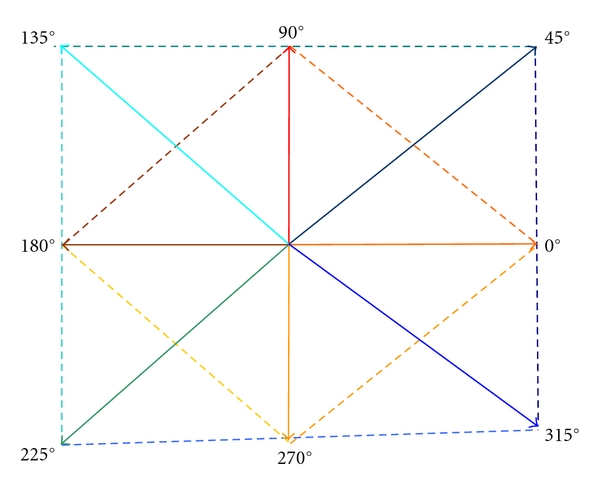
The main directions for the displacement vectors and their combinations in the case of third order GLCM.

For *the third order GLCM*, we considered specific orientations of the displacement vectors. The corresponding three pixels were either collinear, or they formed a right angle triangle (as shown in Figure 1), the current pixel, of coordinates (*x*_1_, *y*_1_), being situated in the central position. Thus, in the case of the collinearity of the pixels, the direction pairs were (0°, 180°), (90°, 270°), (45°, 225°), (135°, 315°), while in the second case, the following direction pairs were considered: (0°, 90°), (90°, 180°), (180°, 270°), (0°, 270°), (45°, 135°), (135°, 225°), (225°, 315°), and (45°, 315°). The values of $\left|\vec{d}x_{i}\right|$ and $\left|\vec{d}y_{i}\right|$ were 0 or 2, with *i* ∈ {1,2}.

**The Implementation of the Fifth Order GLCM**

For the fifth order GLCM, the following groups of directions were taken into account: (0°, 180°, 90°, 270°), respectively, (45°, 225°, 135°, 315°). The current pixel, of coordinates (*x*_1_, *y*_1_), was situated in the central position. The values of $\left|\vec{d}x_{i}\right|$ and $\left|\vec{d}y_{i}\right|$ were 0 or 2, with *i* ∈ {1,2, 3,4}.

**(2) The Cooccurrence Matrix of Edge Orientations**

The generalized cooccurrence matrix (GCM), defined by Davis and Jones in [19], represents the natural extension of the gray level cooccurrence matrix (GLCM), by taking into consideration, instead of the grey levels of the pixels, local features such as edges (points of increased gradient value) or edge orientations detected in the image through specific methods [12].

The edge orientation cooccurrence matrix (EOCM) of order two was defined by Davis and Jones [19] and implemented by Raeth [2] in order to analyze the contour shape of the malignant tumors. We consider the following definition for the edge orientation cooccurrence matrix of order *n*:

(8) $$\begin{array}{cc} & C_{D}\left(o_{1},o_{2},o_{3},\ldots ,o_{n}\right)\\ & \;=\#\{\left(\left(x_{1},y_{1}\right),\left(x_{2},y_{2}\right),\left(x_{3},y_{3}\right),\ldots ,\left(x_{n},y_{n}\right)\right):\\ & \;\;\;\;\text{edge}\_\text{ori}\left(x_{1},y_{1}\right)=o_{1},\text{edge}\_\text{ori}\left(x_{2},y_{2}\right)=o_{2},\ldots ,\\ & \;\;\;\;\text{edge}\_\text{ori}\left(x_{n},y_{n}\right)=o_{n},\left|x_{2}-x_{1}\right|=\left|\vec{d}x_{1}\right|,\;\\ & \;\;\;\;\left|x_{3}-x_{1}\right|=\left|\vec{d}x_{2}\right|,\ldots ,\left|x_{n}-x_{1}\right|=\left|\vec{d}x_{n-1}\right|,\\ & \;\;\;\;\left|y_{2}-y_{1}\right|=\left|\vec{d}y_{1}\right|,\\ & \;\;\;\;\left|y_{3}-y_{1}\right|=\left|\vec{d}y_{2}\right|,\ldots ,\left|y_{n}-y_{1}\right|=\left|\vec{d}y_{n-1}\right|,\\ & \;\;\;\;\mathrm{sgn}\left(\left(x_{2}-x_{1}\right)\left(y_{2}-y_{1}\right)\right)=\mathrm{sgn}\left(\vec{d}x_{1}\cdot \vec{d}y_{1}\right),\ldots ,\\ & \;\;\;\;\;\mathrm{sgn}\left(\left(x_{n}-x_{1}\right)\left(y_{n}-y_{1}\right)\right)=\mathrm{sgn}\left(\vec{d}x_{n-1}\cdot \vec{d}y_{n-1}\right)\}. \end{array}$$

Thus, each element of this matrix is equal with the number of *n*-tuples of pixels with spatial coordinates (*x*_*i*_, *y*_*i*_), *i* ∈ {1,…, *n*}, the values of the edge orientations in these points being *o*_*i*_, *i* ∈ {1,…, *n*}. The spatial relation between the pixels is defined by the set of the displacement vectors, in a similar way with the case of the superior order GLCM. In practice, the EOCM probability matrix was used, being defined in a similar way with the GLCM probability matrix. The edge orientation parameter was computed in each edge point (point of nonzero gradient) by applying the arctangent function on the fraction *G*_*y*_(*x*, *y*)/*G*_*x*_(*x*, *y*), *G*_*y*_ being the vertical image gradient in the point (*x*, *y*) while *G*_*x*_ was the horizontal gradient in the same point. These gradient values were determined by using the Sobel convolution kernel for horizontal and vertical directions [12]. The extended Haralick features—contrast, variance, correlation, energy, and entropy—were defined in the same way as those corresponding to the superior order GLCM, detailed in Appendix B. The second- and third order EOCM were considered in our analysis. The directions of the displacement vectors and their combinations were chosen in a similar way with those corresponding to the case of second- and third order GLCM. The maximum probability parameters were also determined.

#### 2.3.2. The Role of the New Textural Features in Improving the Textural Model of the Malignant Tumors

During the Image Analysis Phase, the old textural features were first computed. Then, the newly defined texture analysis methods were applied, and the corresponding textural features were determined in different conditions, by varying the displacement vector directions and the combinations of these directions. The groups of features corresponding to different values of the intrinsic parameters were assessed separately by powerful classifiers. The discrimination ability of the newly defined textural features was assessed as well, by feature selection methods and appropriate classifiers.

Thus, during the learning phase, feature selection methods were applied in order to estimate the relevance of the textural attributes. First, the new textural features were evaluated individually, by considering only the group of the new textural attributes, obtained for various instances of the intrinsic parameters. Then, the new textural features, corresponding to the most successful configuration of the intrinsic parameters, were considered in combination with the old textural features, in order to assess the increase in accuracy and to derive the final set of relevant textural features by applying the feature selection methods.

In this work, the selection of the relevant textural features was implemented by using the correlation-based feature selection (CFS) method, in combination with genetic search [10], for retaining those textural features that were mostly correlated with the class parameter, and less correlated with the other textural features. For each group of features, a merit was computed:

(9) $$\;\text{Meri}{\text{t}}_{s}=\frac{k\bar{r_{\text{cf}}}}{\sqrt{k+k\left(k-1\right)\bar{r_{\text{ff}}}}}.$$

In (9), Merit_*s*_ is the heuristic merit of the subset *S*, containing *k* features, $\;\bar{r_{\text{cf}}}$ represents the average correlation of the features with the class parameter, while $\bar{r_{\text{ff}}}$ is the average correlation between the features. These correlations were established using the symmetrical uncertainty formula [10]. This method was implemented in combination with genetic search, in order to obtain a complete set of attributes subsets to be analyzed [16].

Another feature selection method, the information gain attribute evaluation, that performed the assessment of the individual attributes was also used. Each attribute was assigned a score based on the information gain between itself and the class:

(10) $$IG_{i}=H\left(C\right)-H\left(C\;|\;A_{i}\right),$$

where *H*(*C*) is the entropy of the class before observing the attribute *A*_*i*_, respectively, *H*(*C* | *A*_*i*_) is the entropy of the class after observing the attribute *A*_*i*_. The amount by which the entropy of the class decreased after observing the attribute *A*_*i*_ revealed the additional information about the class and constituted the information gain which was due to the attribute *A*_*i*_.

During the Validation Phase, we adopted the classifiers of multilayer perceptron (MLP) [11] and support vector machines (SVM) [11], as they led to the best results in our former experiments [6]. The AdaBoost combination scheme [11], having the methods of MLP and SVM as basic classifiers, was also implemented.

## 3. Results and Discussions

### 3.1. Description of the Experiments

We considered a number of 300 patients suffering from HCC, 100 patients with hemangioma, 70 patients with colonic tumors, and 70 patients with inflammatory bowel diseases. Each of the considered type of disease corresponded to a class in the training, respectively, test set. These classes were combined in equal proportions inside the dataset. For each patient, three to five images were considered, acquired for various orientations of the transducer. The images were acquired using a Logiq 7 ultrasound machine, at the frequency of 5.5 MHz, the depth being 16 cm. During the image analysis phase, rectangular regions of interest, having 50×50 pixels in size, were selected on each type of analyzed tissue. After performing noise reduction using an averaging filter, the old textural features were computed independently of the orientation and illumination conditions. The new textural features were computed for various values of the parameters, as described previously. The values of the textural features, for each region of interest, were stored in specific files, for further analysis.

During the learning phase, the feature selection and classification experiments were performed using the methods of the Weka 3.5 library [16]. For feature selection, the method of correlation-based feature selection (CFS) was used, in conjunction with genetic search. For the genetic search method, the seed had the value 1, the crossover probability was 0.6, the mutation probability was 0.033, the population size was 20, and the number of generations to be evaluated was 20. The feature selection method that performed feature evaluation based on the information gain of the attributes with respect to the class, the information gain attribute evaluation method of the Weka 3.5 library, was also implemented during the learning phase, in conjunction with the Ranker search method.

During the validation phase, the Weka 3.5 versions of the support vector machines (SVM) method, the multilayer perceptron (MLP) classifier, and the AdaBoost combination scheme that used the SVM and MLP classifiers as basic learners were implemented. In the case of the SVM classifier, John's Platt sequential minimal optimization (SMO) algorithm for training a support vector classifier was used [16]. The polynomial kernel of 3rd degree, which provided the best result in our former experiments [6], was adopted for the SVM method. In the case of the MLP method, the learning rate was 0.2 in order to obtain a refined learning process and to avoid overtraining. The momentum was 0.8 in order to achieve a fast crossing over the plane areas of the learning surface. The number of nodes from the hidden layer was the arithmetic mean between the number of the input features and the number of classes. The AdaBoost *M*1 combination procedure of Weka 3.5, with 10 iterations, was implemented as well. The stratified cross-validation method strategy with 5 folds was used for classifier evaluation. Thus, for each iteration of the cross-validation method, the training set was formed by considering 80% of the data instances, while the test set consisted of 20% of the data instances.

### 3.2. Results

#### 3.2.1. Performing the Differentiation between the HCC Tumor and the Cirrhotic Liver Parenchyma on Which the Tumor Evolved

**Figure 2:**
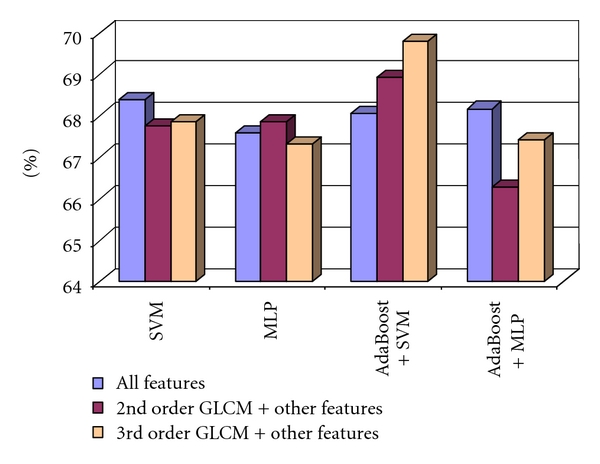
The evaluation of the 3rd order GLCM performances compared with those of the 2nd order GLCM, when considering the averaged values for all the directions, in the case of the differentiation between HCC and cirrhotic parenchyma.

Figure 2 illustrates the classification performance achieved by using the group of third order GLCM textural features, in comparison with that achieved by using the group of second order GLCM textural features. In this situation, the second- and the third order GLCM features were averaged after considering all the adopted directions. The following feature sets were taken into account: the second order GLCM features combined with the other textural features, represented with red color in Figure 2; the third order GLCM features combined with the other textural features, represented with yellow; the entire set of textural features, consisting of the third order GLCM features, the second order GLCM features, and the other textural features, represented with blue. Figure 2 illustrates the recognition rates obtained for these sets of features using the adopted classifiers. From Figure 2, it results that the third order GLCM led to a better classification performance than the second order GLCM in most of the situations. However, the best results were obtained when considering all the textural features, involving the features derived from both types of GLCM, combined with the other textural features.

**Figure 3:**
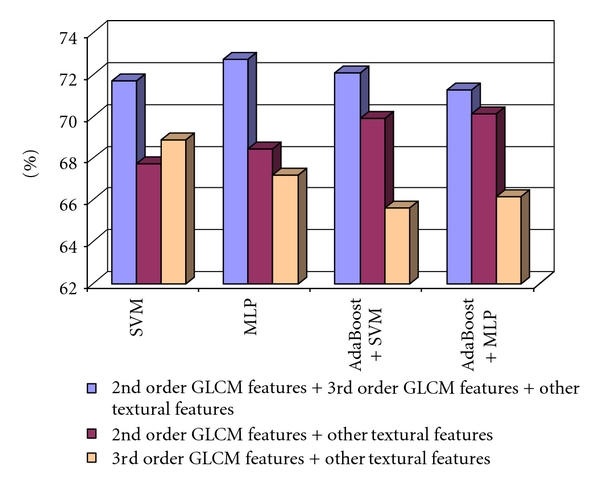
The recognition rate obtained when considering the 2nd order GLCM features combined with the other textural features, the 3rd-order GLCM textural features and the other textural features, respectively the 2nd-order GLCM, the 3rd-order GLCM, and the other textural features, in the case of the differentiation between HCC and cirrhotic parenchyma.

Further experimental steps consisted of the assessment of the combinations between the directions of the displacement vectors, in order to detect the combination that leads to the best classification accuracy. The best classification accuracy was obtained when considering only the (0°, 270°) combination of directions. When combining the third order GLCM Haralick features obtained for the (0°, 270°) directions with all the other textural features (including the second order GLCM features), we obtained the best classification performance.  Figure 3 illustrates the results concerning the improvement in the recognition rate.

**Figure 4:**
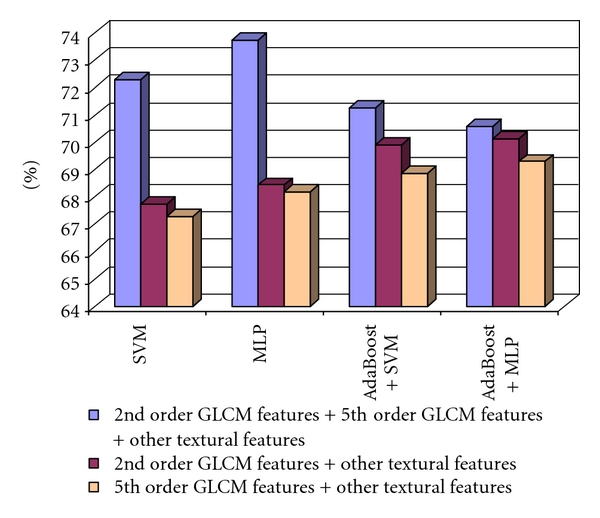
The recognition rate obtained when considering the 2nd order GLCM features combined with the other textural features, the 5th-order GLCM textural features and the other textural features, respectively, the 2nd order GLCM, the 5th-order GLCM, and the other textural features, in the case of the differentiation between HCC and cirrhotic parenchyma.

Considering the case of the fifth order GLCM, the best classification results were obtained for the directions (0°, 180°, 90°, and 270°). The classification accuracy, resulted after combining the fifth order GLCM parameters obtained for the above group of displacement vector directions, combined with the other textural feature, is depicted in Figure 4.

As we can notice, the classification accuracy was more increased when considering all the textural features, than in the case when we used only the old textural features, achieving the maximum value of 73.75% in the case of the multilayer perceptron (MLP) classifier. We also considered the features derived from the second- and third order cooccurrence matrices of edge orientations. The second order cooccurrence matrix of edge orientations (EOCM) was computed for the directions 0°, 45°, 90°, and 135°, and the values of the resulted features were averaged. The second order EOCM features, combined with the old textural features, led to a recognition rate of 75%, for the AdaBoost metaclassifier that used MLP as a basic classifier, and to a value of AUC above 80%.

The third order EOCM matrix was computed in a similar way with the third order GLCM matrix. The relevant textural features, obtained after applying the methods of correlation-based feature selection (CFS) and information gain attribute evaluation, indicated the prevalence of the (45°, 315°) and (0°, 90) pairs of the displacement vector directions. After providing the extended Haralick features to the inputs of the SVM and MLP classifiers, the (0°, 90°) pair of directions provided the best results, as illustrated in Table 1.

**Figure 5:**
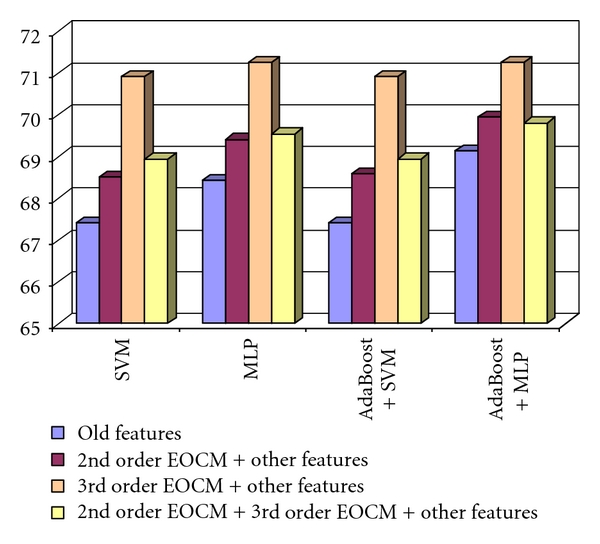
The recognition rate obtained when considering the 2nd order EOCM features combined with the old textural features, the 3rd order EOCM textural features and the old textural features, respectively, the 2nd order EOCM, the 3rd order EOCM, and the old textural features, in the case of the differentiation between HCC and cirrhotic parenchyma.

After combining the second- and third order EOCM features with the old textural features, we obtained a recognition rate situated above 71%. The combinations between the second order EOCM textural features and the old textural features, respectively, between the third order EOCM textural features and the old textural features, always led to an accuracy improvement compared with the case when only the old features were used. The combination between the second order EOCM textural features, the third order EOCM textural features, and the old textural features led, in most of the situations, to an accuracy improvement, compared with the cases when only the old textural features, or the combination between the second order EOCM textural features and the old textural features were used. The combination between the third order EOCM textural features and the old textural features provided the best recognition rates, in all the situations. These results can be visualized in Figure 5.

The final set of the relevant textural features, for the case of differentiation between HCC and cirrhotic parenchyma, resulted after performing feature selection on the group formed by the old textural features, by the third order GLCM features, by the fifth order GLCM features, by the second order EOCM features, and by the third order EOCM features. This set consisted of the union between the features selected by the CFS method, and those selected by the method of information gain attribute evaluation. After we provided the values of the relevant textural features at the classifier inputs, we obtained a recognition rate of almost 78% for the MLP classifier and an AUC of above 82% for the same classifier, as we can observe in Table 2.

**Figure 6:**
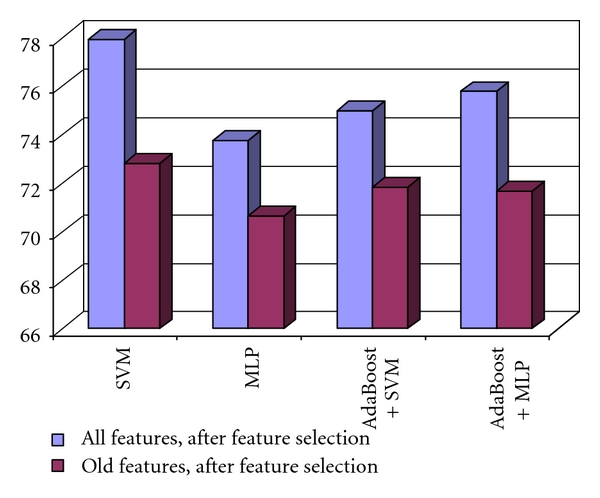
The increase of the recognition rate, obtained when using the new relevant textural features, compared with those obtained when using the old relevant textural features in the case of the differentiation between HCC and cirrhotic parenchyma.

The increase in the recognition rate, obtained by using the final set of the new and old relevant textural features, compared with the accuracy due only to the old relevant textural features is depicted in Figure 6. Thus, an increase in accuracy from 71% to almost 78% was achieved, due to the new textural features, in the case of differentiation between HCC and the cirrhotic parenchyma on which the tumor evolved.

**The Relevant Textural Features for the Differentiation between HCC and the Cirrhotic Parenchyma on Which HCC Had Evolved**

After performing feature selection using the CFS and information gain attribute evaluation methods, the most important textural features contained in the union of the two feature subsets were the mean of the grey levels, indicating differences in echogenicity between the HCC tumor and the cirrhotic liver parenchyma, because, as it is well known, the HCC tumor, in advanced evolution phases, is hyperechogenic in most of the cases. The third- and fifth order GLCM correlation and the autocorrelation index indicated differences in granularity between the HCC tumor and the cirrhotic liver parenchyma. The second- and third order GLCM homogeneity, second- and third order GLCM contrast, and the fifth order GLCM variance, provided information about the inhomogeneous aspect of the tumor tissue. The fifth order GLCM energy and entropy, the third order EOCM entropy, and the entropy computed after applying the Wavelet transform at the first level and at the second level, on the low-low component, were increased in the case of the tumor tissue, indicating its chaotic structure. The edge orientation variability and the frequency of the Laws textural microstructures indicated the complexity of the malignant tumor, which was constituted by multiple types of tissues.

**The Values of the Maximum Probability Parameters**

In the case of the third order GLCM, the maximum value of the probability for a given combination of three grey levels to appear within the considered class of tissue was around 0.01 in both cases of HCC and cirrhotic parenchyma on which HCC had evolved. This result was derived as an arithmetic mean of the maximum probability parameters computed on all the images belonging to the 300 patients included in the dataset. This probability was decreased in comparison with the same parameter computed in the case of the second order GLCM, when the mean value of the maximum probability was 0.05. The experimental results also revealed that groups of three hypoechogenic pixels (57, 57, 57) corresponding to pure tumor regions with active growth, or to regions affected by necrosis, appeared frequently inside HCC. These values appeared rarely inside the cirrhotic parenchyma and inside the benign liver tumors.

In the case of the fifth order GLCM, the probability for a given combination of five gray level values to occur in the region of interest was computed separately for HCC and the cirrhotic parenchyma, for the (0°, 180°, 90°, 270) group of displacement vector directions, which provided the best accuracy results. The maximum probability had the value of 0.0035 in the case of HCC, respectively, 0.0036 in the case of the cirrhotic parenchyma. Thus, the probability was higher in the case of the cirrhotic parenchyma and lower in the case of HCC. This was a normal result, if we take into consideration the chaotic structure of the HCC tissue. For the second order EOCM, the maximum probability for a pair of two edge orientations to appear inside the tissue of the cirrhotic parenchyma was 0.132 while the value of the same parameter in the case of HCC was 0.131. This also emphasized the chaotic character of the HCC tissue, and the more regular character of the cirrhotic parenchyma. The most frequently met pair of two edge orientation values was (0°, 89°) inside both HCC and cirrhotic parenchyma regions, corresponding to the directions of the liver tissue fibers and of the separating walls. For the third order EOCM, the maximum probability for a combination of three values of the edge orientation feature to appear within the HCC tissue was 0.00115 while in the case of the cirrhotic parenchyma, the value of this parameter was 0.00129. The most frequently met combination of three edge orientation values was (90°, 45°, 45°) inside the HCC tissue, respectively, (45°, 0°, 45°) inside the cirrhotic parenchyma on which HCC had evolved.

#### 3.2.2. Performing the Differentiation between HCC and the Benign Liver Tumors

The third order GLCM was also experimented in the case of the differentiation between HCC and the benign liver tumors. After performing relevant feature selection using the CFS and information gain attribute evaluation methods, the features corresponding to the (45°, 225°), (45°, 135°), respectively, (0°, 90°) direction pairs appeared to be relevant. After applying the SVM and MLP classifiers for the final assessment of the efficiency of the displacement vector direction pairs, we noticed that the (0°, 90°) combination provided the best results.

**Figure 7:**
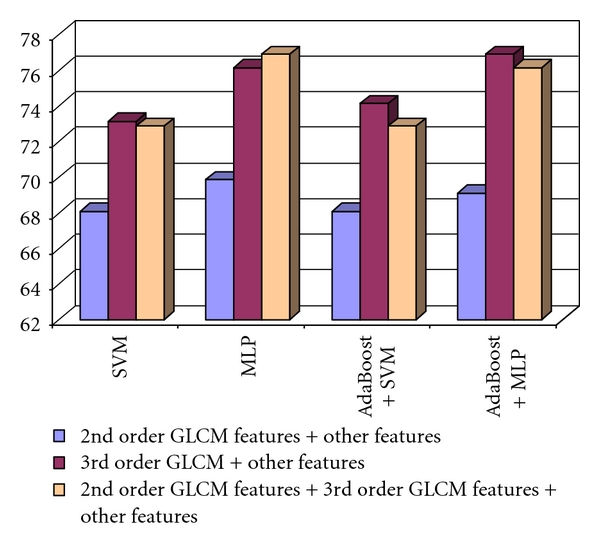
The recognition rates obtained when considering the group of 2nd order GLCM features combined with the other textural features, those of the 3rd order GLCM textural features together with the other textural features, respectively, the groups formed by 2nd order GLCM, the 3rd order GLCM, and the other textural features in the case of the differentiation between HCC and the benign liver tumors.

The comparison between the recognition rates obtained in the cases of using the second order GLCM textural features combined with the other textural features, the third order GLCM textural features combined with the other textural features, respectively, the second order GLCM, the third order GLCM and the other textural features, is illustrated in Figure 7. As we can notice, the combination between the third order GLCM textural features and the other textural features outperformed the two other groups of features in most of the situations. The best recognition rate, of 76.88%, was obtained in the case of applying the AdaBoost combination scheme that used the MLP classifier as a basic learner.

**Figure 8:**
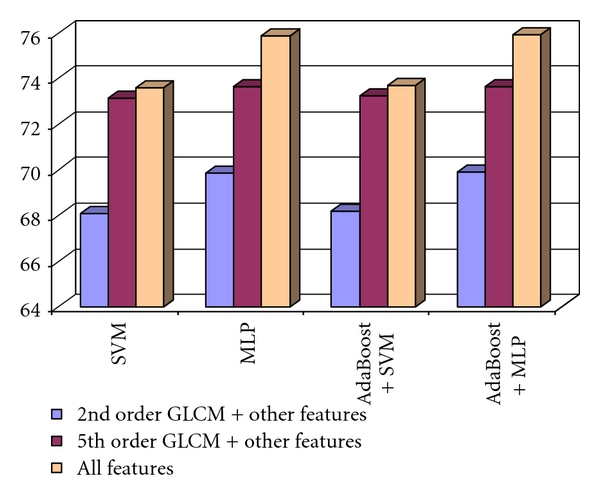
The recognition rate obtained when considering the 2nd order GLCM features and the other textural features, the 5th order GLCM textural features and the other textural features, respectively the 2nd order GLCM, the 5th order GLCM, and the other textural features in the case of the differentiation between HCC and the benign liver tumors.

Concerning the fifth order GLCM, the assessment of the two considered directions groups revealed that using all the textural features, provided by both versions of the fifth order GLCM, led to the best results. After the combination of the fifth order GLCM features with the other textural features (except the second order GLCM features), the recognition rates were always higher than in the case of using only the second order GLCM features and the other textural features. The best recognition rate was achieved when considering both the second order GLCM and the fifth order GLCM textural features, together with the old textural features. This result can be visualized in Figure 8.

The highest values of the accuracy parameters were obtained for the combination between the second order GLCM textural features, the fifth order GLCM textural features and the old textural features, in the case of the MLP classifier, respectively, AdaBoost metaclassifier that used the MLP method as a basic classifier. In the latter case, the best value of the recognition rate, of 75.90%, was achieved.

**Figure 9:**
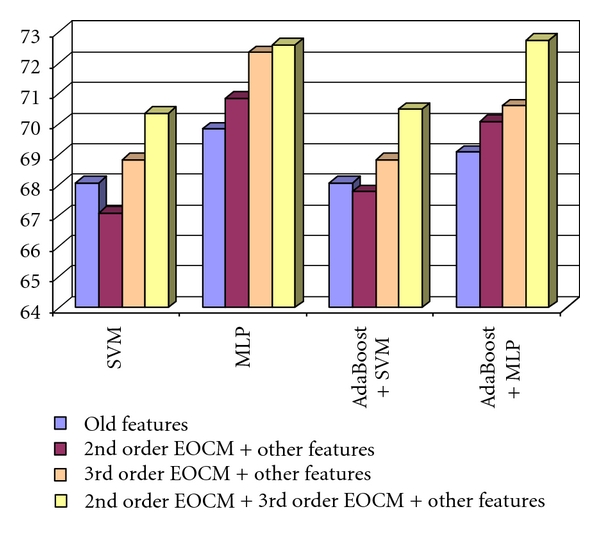
The comparison of the recognition rates obtained by combining the 2nd and 3rd order EOCM features with the old textural features in the case of the differentiation between HCC and the benign liver tumors.

The second order EOCM textural features were computed in a similar way as in the case of differentiating between the HCC tumor and the cirrhotic parenchyma. Concerning the textural features derived from the third order EOCM matrix, the CFS and information gain feature evaluation methods were applied again for relevant feature selection. The two sets of important textural features emphasized the frequency of those attributes corresponding to the (0°, 90°), (0°, 180°), and (0°, 270°) combinations of directions. After the assessment through the MLP and SVM classifiers, the (0°, 270°) direction group was found to be the best. The results obtained after combining the second- and third order EOCM textural features, corresponding to the (0°, 270°) pair of displacement vector directions, with the old textural features are illustrated in Figure 9. It results, from Figure 9, that the best recognition rates were achieved when combining both the second- and third order EOCM features with the old textural features. Also, the combination between the third order EOCM textural features and the old textural features led to a better recognition rate that in the cases when using the combination between the second order EOCM features and the old textural features, respectively, only the old textural features.

The best recognition rate, of 72.75%, was achieved in the case of combining the third order EOCM features with the old textural features, and using the AdaBoost combination scheme in conjunction with the MLP method. The old textural features, the features derived from the third- and fifth order GLCM matrix, respectively from the second- and third order EOCM matrix, were finally combined and a single group of textural features was obtained. After applying the CFS and information gain attribute evaluation methods, the final set of relevant textural features for differentiating HCC from hemangioma resulted as the union between the two resulted feature subsets. The values of the accuracy parameters resulted after providing the final set of relevant textural features at the classifiers inputs is illustrated in Table 3. As we can notice, a recognition rate of 83.66% was obtained in the case of AdaBoost combination scheme that used the MLP as basic classifier, and also an increased AUC, of 89.9%, was obtained for the MLP classifier. We also remark on the increased specificity (TN rate), always situated above 86%.

**Figure 10:**
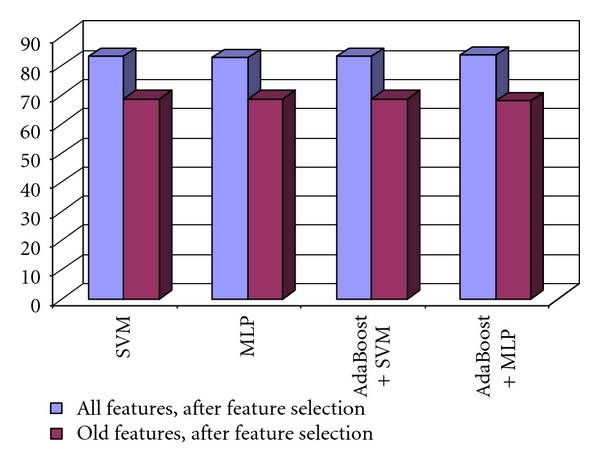
Comparison between the recognition rates obtained by using the entire set of relevant textural features, respectively, the old set of relevant textural features features in the case of the differentiation between HCC and the benign liver tumors.

In Figure 10, we can also visualize the increase in the recognition rate, due to the new derived textural features, compared with that obtained by providing the original set of relevant textural features at the classifier inputs. Thus, an accuracy increase, from 70%, to 80% can be noticed.

**The Relevant Textural Features for the Differentiation between HCC and the Benign Tumors**

In the final set of relevant textural features, we noticed the presence of the second-third-, and fifth order GLCM features, which played an important role in the differentiation between the HCC tumor and the benign tumors. Features like the third order GLCM homogeneity, third- and fifth order GLCM contrast, respectively, the third order GLCM variance, emphasized the difference in homogeneity and complexity in the grey level structure between the HCC tumor and the benign tumors. The fifth order GLCM correlation, together with the autocorrelation index, revealed differences in granularity between the malignant tumors and the benign tumors. The energy and entropy of the third order EOCM matrix were important as well, putting into evidence the uniformity of edge orientations present in the case of the benign tumors, and the lack of it in the case of the malignant tumors, where the entropy parameter has higher values. The features computed after applying the Wavelet transform were also important. The entropy was more emphasized at the second level of resolution in the case of HCC. The spot textural microstructures, determined after applying the Laws convolution filters, were frequently met inside HCC and sparsely met inside the benign tumor tissue. Thus, the spots contributed to the differentiation of the HCC tumor from the cirrhotic liver parenchyma and from the benign tumors as well.

**The Values of the Maximum Probability Parameters**

The value of the maximum probability parameter determined in the case of the third order GLCM, equivalent with the probability to encounter a combination of three grey levels inside the benign liver tumors, was 0.05, which was higher than the value of the same parameter computed in the case of HCC (0.01), emphasizing, once more, the chaotic structure of the malignant tumor tissue. The average value of the maximum probability parameter computed in the case of the fifth order GLCM matrix was 0.007 for the class of benign tumors, being again more increased than the value of the same parameter computed in the case of HCC, 0.003. Groups of three hypoechoic pixel values (53, 53, 53) were frequently encountered inside the benign tumors, corresponding to vascular lakes. In the case of the second order EOCM, the maximum probability for a pair of two edge orientation values to occur inside the benign tumor region was 0.138, this being larger than the value of the same parameter computed in the case of HCC (0.131). The pair of edge orientation values that most often appeared inside the benign tumor regions was (0°, 89°), being similar with the edge orientation pair that was met in the case of HCC and cirrhotic parenchyma. The average value of the maximum probability parameter, computed inside the third order EOCM matrix in the case of the benign liver tumors, was 0.0021, being more increased than the same value obtained in the case of the HCC tumor, of 0.0013. The most frequently met combination of three edge orientation values inside the benign tissue was (90°, 90°, 90°), denoting the more regular structure of the tissue.

#### 3.2.3. Performing the Differentiation between the Colorectal Tumors and the Inflammatory Bowel Diseases (IBD)

**Figure 11:**
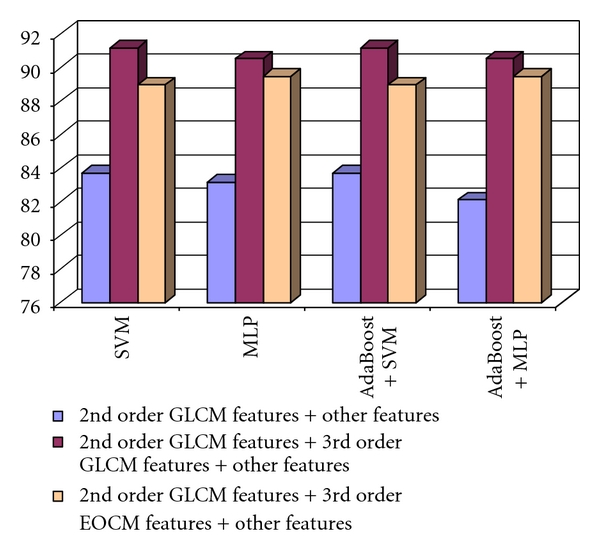
The recognition rate obtained when considering the 2nd order GLCM features and the other textural features, the 2nd order GLCM features, the 3rd order GLCM, and the other textural features, respectively, the 2nd order GLCM features, the 3rd order EOCM features, and the other textural features in the case of the differentiation between the colorectal tumors and IBD.

In the case of the comparison between the colo-rectal tumors and the inflammatory bowel diseases, the best improvement in the classification accuracy was provided by the textural features derived from the third order GLCM, respectively, by those resulted from the third order EOCM. The best combination of displacement vector directions was (0°, 270°) in the case of the third order GLCM, and (0°, 180°) in the case of the third order EOCM. The comparison between the recognition rates obtained in these cases, and in the case of the original textural features, is illustrated in Figure 11. The combination between the third order GLCM textural features, the second order GLCM textural features, and the other textural features always provided the best recognition rate, situated above 90%. The combination between the second order GLCM features, the third order EOCM features, and the old textural features also provided an increase in accuracy, compared with the set of old textural features, in all the situations.

**Figure 12:**
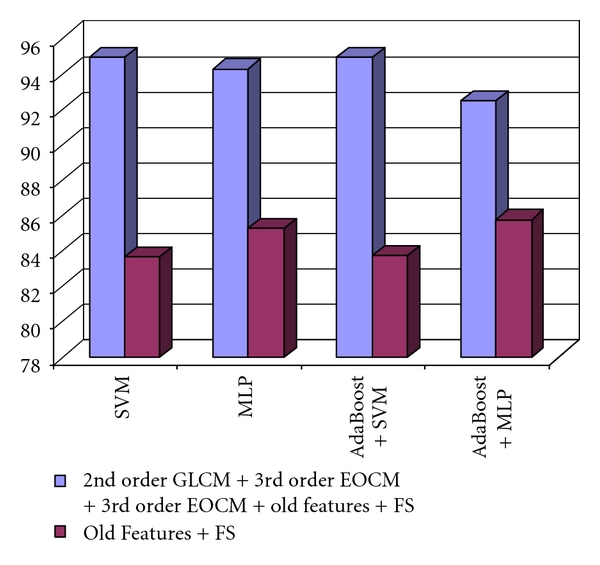
Comparison between the recognition rates obtained by using the entire set of relevant textural features, respectively, the old set of relevant textural features in the case of the differentiation between the colorectal tumors and IBD.

Finally, the second order GLCM textural features, the third order GLCM textural features, the third order EOCM textural features, and the other textural features were combined, and then the relevant textural features were selected. The final set of relevant textural features resulted after performing the union operation between the subsets of important textural features provided by each of the feature selection methods. The comparison between the recognition rates obtained by using the final set of relevant textural features, respectively, the initial set of relevant textural features, obtained by considering only the old textural features, is depicted in Figure 12. An increase in accuracy from 85% to almost 94% can be noticed.

The values of all the considered accuracy parameters, in the case of the final set of relevant textural features, are illustrated in Table 4. The best recognition rate, of 94.93%, was obtained in the case of the SVM classifier, respectively, in the case of AdaBoost combination scheme that used the SVM as basic classifier. We also noticed the increased value of AUC, of 98.3%, obtained in the case of the MLP classifier.

**The Relevant Textural Features for the Differentiation between the Colorectal Tumors and the Inflammatory Bowel Diseases**

The third order GLCM homogeneity, as well as the third order EOCM homogeneity resulted to be important in order to distinguish between the colo-rectal tumors and the IBD, due to the heterogeneous structure of the tumor tissue. The energy and the entropy features were also relevant when derived from the second order GLCM, from the third order GLCM, as well as from the third order EOCM, emphasizing the chaotic structure and the irregular aspect of the colo-rectal tumor tissue, respectively, the more regular aspect of the bowel wall that correspond to the IBD case. The entropy computed at the first level after applying the wavelet transform was also important in this context. Concerning the textural microstructures obtained after applying the Laws convolution filters, the spots and the waves appeared to be more emphasized in the tumor region, suggesting the presence of severe fibrosis and also the complex structure of the tumor.

**The Values of the Maximum Probability Parameters**

The maximum probability for a pair of three grey level values to occur in the region of interest was 0.009 inside the colo-rectal tumors, respectively, 0.014 on the bowel wall affected by IBD. Groups of hyperechogenic values, corresponding to tissue regions strongly affected by fibrosis, were often met inside the colo-rectal tumors. The maximum probability for a pair of three edge orientation values to occur was 0.0013 inside the colo-rectal tumors and 0.0026 on the bowel wall affected by IBD. The most frequent group of edge orientations that appeared inside the colo-rectal tumor was (90°, 45°, 90°), respectively, on the bowel wall, this group was (90°, 90°, 90°). This fact confirms the more complex aspect of the bowel wall that exists in the presence of the colo-rectal tumor.

### 3.3. Discussions

As the experiments revealed, the new implemented methods for texture analysis, based on the superior order cooccurrence matrices led to a considerable accuracy increase and to a better emphasis of the malignant tumors characteristics, in comparison with those of the benign tumors and the tissue of the visually similar diseases. Concerning the orientations of the displacement vectors, the combinations between the horizontal and vertical directions led to the best accuracy results. The best orientations of the displacement vectors were also parallel or perpendicular on the direction of the ultrasound signal propagation. The combination between the old textural features, the third- and fifth order GLCM features, respectively, the second- and third order EOCM features, followed by feature selection, led to an increase of the recognition rate from 70% to 80% in both cases of differentiation between HCC and cirrhotic liver parenchyma, and between HCC and the benign tumors, respectively, to an accuracy improvement from 80% to 90% in the case of the colo-rectal tumor recognition. The probability of a certain combination of grey levels to appear inside the tissue, determined by the maximum probability of the grey level cooccurrence matrix, had higher values in the case of the benign tumors and of the visually similar tissues, and lower values in the case of the malignant tumors, putting into evidence the complex structure of the malignant tissue. In the case of the cooccurrence matrix of edge orientations, the same situation appeared. Also, the third order EOCM energy was higher inside the benign tumors and lower inside the malignant tumors, which indicated an increased uniformity of the edge orientations inside the benign tumors and the irregularity of the values of this feature in the case of the malignant tumors. The most frequently met edge orientation values inside the malignant tumors and the cirrhotic parenchyma are 0°, 45°, and 90°, while in the case of the benign tumors and of the bowel wall affected by IBD, only the 0°, 90° values were met more often. This emphasized the complexity of the malignant tumors, respectively, of the tissue affected by diseases that precedes cancer, such as cirrhosis. We also noticed that the value of the maximum probability parameter decreased while the order of the cooccurrence matrix increased. However, the cooccurrence matrices of order *n*, *n* > 2 always led to accuracy improvements and to a more refined characterization of the analyzed tissues, as shown in the experiments section. Concerning the relevant textural features that differentiated the malignant tumors from the other kinds of tissues, the homogeneity, the variance, and the contrast computed from the cooccurrence matrices indicated the heterogeneous structure of the malignant tumor tissue. The correlation, together with the autocorrelation index, emphasized a difference in granularity between the restructuring areas of the malignant tumors and the tissue zones corresponding to less aggressive diseases. The entropy computed after applying the wavelet transform revealed the presence of the chaotic character at multiple resolutions, in the case of the malignant tumors. The textural microstructures, determined after applying the Laws convolution filters, were also important in order to distinguish the malignant tumors from the visually similar tissues, emphasizing the complexity of the tissue affected by malignancy.

## 4. Conclusions and Future Work

The superior order grey level cooccurrence matrices, as well as the edge orientation cooccurrence matrices of superior order, led to an improvement of the classification performance, in comparison with the case when only the old textural features were used. The probability for a certain combination of gray levels or edge orientations to occur in the region of interest was lower in the case of the tumor tissue and higher in the case of the visually similar tissues. This fact reflected the irregular structure of the malignant tissues. The value of the maximum probability parameter decreased while the order *n* of the superior matrix increased, as the number of possible combinations of feature values increased and the evaluation became more refined. The final set of relevant textural features revealed, in each case, the presence of the new textural features, derived from superior order matrices, and emphasized the inhomogeneous, complex, chaotic structure of the malignant tumor tissue. The smaller classification accuracy obtained in the case of HCC tumor recognition is due mainly to the variations in the aspect of the HCC tumor, and also to the small differences that exist between the HCC and cirrhotic parenchyma tissues, both diseases involving a restructuring process. In the case of colorectal tumor recognition, the classes were more homogeneous, so the classification accuracy was higher. In our future work, we aim to divide the HCC tumor into subclasses and to improve the classification accuracy through multiclass classification. The specific groups of grey levels or edge orientation values that appeared inside each subclass of malignant tumors will be further analyzed and their correspondence with the tissue microstructures will be established. We will also implement more complex classifier combination schemes, such as stacking, in order to improve the automatic diagnosis performance. The computation of the extended Haralick features at multiple resolutions is a future research objective as well.

## Figures and Tables

**Figure 13:**
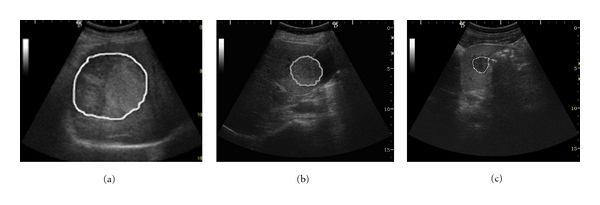
The malignant and benign liver tumors: (a) hepatocellular carcinoma, encephalic form, (b) hemangioma; (c) focal nodular hyperplasia.

**Figure 14:**
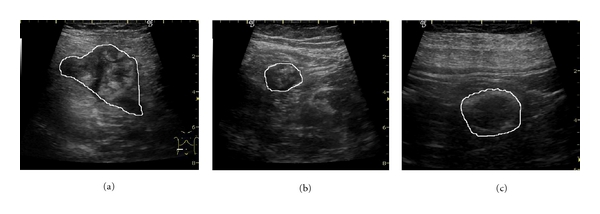
The colo-rectal tumors and the inflammatory bowel diseases: (a) colo-rectal tumor; (b) Crohn's disease; (c) ulcero-hemorrhagic recto-colitis.

**Figure 15:**
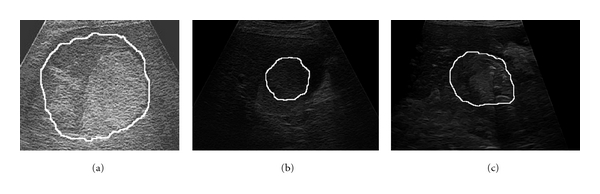
The properties of the edges inside the tumoral tissue (a) edges inside HCC; (b) edges inside hemangioma; (c) edges inside the colo-rectal tumor.

**Figure 16:**
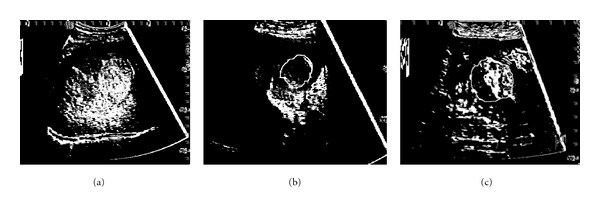
The spot textural microstructures inside the tumoral tissue (a) HCC image after the detection of the spot microstructures; (b) hemangioma image after the detection of the spot microstructures; (c) colo-rectal tumor after the detection of the spot microstructures.

**Table 1:** The classification performance obtained by using the third order EOCM features for the most important combinations of displacement vector directions.

Comb. of dir.	Classif. meth.	Recog. rate	TP rate	TN rate	AuC	Time
(45°, 315°)	SVM	65.27%	48.9%	84.6%	63.3%	129.25 s
	MLP	61.176%	58.2%	64.2%	63.9%	19.08 s
(0°, 90°)	SVM	65.81%	46.6%	85%	65.8%	157.95 s
	MLP	62.51%	60.9%	64.2%	67.6%	17.74 s

**Table 2:** The accuracy results obtained by considering the final set of relevant textural features.

Classifier	Recognition rate	TP rate	TN rate	AUC	Time
SVM	77.94%	78.3%	77.6%	77.9%	78.21 s
MLP	73.75%	70.1%	77.4%	82.3%	54.32 s
AdaBoost + SVM	75%	79%	71%	78.6%	86.25 s
AdaBoost + MLP	75.79%	74.6%	76.7%	82%	87.55 s

**Table 3:** The values of the accuracy parameters obtained by using the final set of relevant textural features appropriate for the differentiation between HCC and the benign liver tumors.

Classifier	Recognition rate	TP rate	TN rate	AUC	Time
SVM	83.16%	78.9%	87.4%	83.2%	81.32 s
MLP	82.66%	78.4%	86.9%	89.9%	62.27 s
AdaBoost + SVM	83.21%	79%	88.3%	83.9%	89.22 s
AdaBoost + MLP	83.66%	80.4%	86.9%	84.3%	89.16 s

**Table 4:** The values of the accuracy parameters obtained by using the final set of relevant textural features appropriate for the differentiation between the colo-rectal tumors and the inflammatory bowel diseases.

Classifier	Recognition rate	TP rate	TN rate	AUC	Time
SVM	94.93%	94.9%	94.9%	94.9%	77.18 s
MLP	94.3%	93.9%	94.1%	98.3%	64.28 s
AdaBoost + SVM	94.93%	94.9%	94.9%	94.9%	85.34 s
AdaBoost + MLP	92.50%	91.1%	89.9%	97.7%	83.11 s

## Appendices

### A. The Hepatocellular Carcinoma and the Colonic Tumors: Medical Considerations and Visual Aspect in Ultrasound Images

The hepatocellular carcinoma (HCC) is the most frequent malignant liver tumor, representing 75% of the liver cancer cases, besides hepatoblastoma (7%), cholangiocarcinoma, and cystadenocarcinoma (6%). The most relevant oncogenic agent for HCC development is the chronic viral infection with the hepatitis B virus (HBV), or hepatitis C virus (HCV), the next evolution phase, preceding HCC, being cirrhosis [1]. HCC evolves from cirrhosis, after a restructuring phase, at the end of which dysplastic nodules (future malignant tumors) result. Concerning the visual aspect in ultrasound images, in incipient phase, HCC appears like a small region having a different texture than the other parts of the tissue and a diameter of about 1.5 cm to 2 cm. In the case of an evolved HCC, the essential textural attribute is that of heterogeneity, due to the coexistence of fatty regions, of regions with necrosis, fibrosis, and, respectively, active growths. HCC is also characterized through a complex structure of vessels [1]. Hemangioma is the most frequent form of benign liver tumors, consisting of a mass of abnormal blood vessels. Up to 5 percent of adults in the United States have small hemangiomas in their liver. Concerning the aspect in ultrasound images, most of the hemangiomas are isoechogenic and homogeneous [20]. The focal nodular hyperplasia is another frequent benign liver tumor. The necrosis and hemorrhage are rarely met inside these structures. The histological studies detected the existence of some fibrous bands, of multiple biliary ducts, and of some fibrous scars, of stellar shape. In ultrasound images, the FNH tumors appear as isoechogenic or hypoechogenic lesions, having a homogeneous aspect, but containing a small, stellar scar inside [20]. The colo-rectal tumors represent a frequent disease for the population of the developed countries, being the third cause of cancer-related death in the Western world. They arise from the adenomatous polyps which are present on the bowel wall. Like every tumor, they are characterized by the heterogeneity of the tissue structure and by the complexity and irregularity of the vessel structure [5]. In ultrasound images, they have an inhomogeneous, mixed aspect, the parietal delimitation being linear, but interrupted by the tumor invasion. The adenopathy could also be present inside the tumor, having rounded shape. Although distinct from IBD, they share a lot of characteristics with the latter, like wall thickening and increased vascularity [21]. Some eloquent examples of all the described affections can be visualized in Figure 13.

In Figure 13, the liver tumors are illustrated: an instance of HCC in focal encephalic form, evolved phase; an instance of hemangioma benign tumor; an instance of the FNH benign tumor. In Figure 14, an example of a colo-rectal tumor is illustrated. The shape modifications of the bowel wall, due to the colo-rectal tumor, are also visible in Figure 14(a). The inflammatory bowel diseases-Crohn's disease and ulcerohemorrhagic rectocolitis are also depicted.

### B. Description of the Superior Order Haralick Features

The contrast of order *n* for the generalized cooccurrence matrices was computed as follows:

(B.1) $$\begin{array}{cc} \text{Contrast} & =\overset{M-1}{\underset{f_{1}=0}{\sum }}\;\overset{M-1}{\underset{f_{2}=0}{\sum }}\cdots \overset{M-1}{\underset{f_{n}=0}{\sum }}\left(\overset{n-1}{\underset{u=1}{\sum }}\;\overset{n}{\underset{v=u+1}{\sum }}\text{dif}{\left(f_{u},f_{v}\right)}^{2}\right)\\ & \;*p\left(f_{1},f_{2},\ldots ,f_{n}\right). \end{array}$$

In (B.1), *f*_1_, *f*_2_,…, *f*_*n*_ are the values of the descriptors for the considered features: in the case of the GLCM of order *n*, they are the grey levels of the pixels, while in the case of superior order EOCM, the values of the edge orientations are taken into account; dif(*f*_*u*_, *f*_*v*_) is the absolute value of the difference between any two values of the local features involved in a certain *n*-tuple that gives the coordinates of a superior order matrix element. *M* is the maximum value that a certain feature (grey value of edge orientation) can achieve. The contrast, also called dissimilarity, estimates the difference that exists, in the region of interest, between the values of the considered local features, corresponding to pixels that are in a spatial relation defined by the displacement vectors. It measures the local variations in the grey level cooccurrence matrix.

Concerning the other measures, we defined the following mathematical formulas:

(B.2) $$\begin{array}{cc} \text{Entropy} & =\overset{M-1}{\underset{f_{1}=0}{\sum }}\overset{M-1}{\underset{f_{2}=0}{\sum }}\cdots \overset{M-1}{\underset{f_{n}=0}{\sum }}p\left(f_{1},f_{2},\ldots ,f_{n}\right)\\ & \;\times \left(-\ln p\left(f_{1},f_{2},\ldots f_{n}\right)\right). \end{array}$$

The entropy expresses the disorder of the texture, with respect to the considered feature (grey level or edge orientation value), the uncertainty for a certain combination of *n* values (*f*_1_, *f*_2_,…, *f*_*n*_) to appear,

(B.3) $$\text{Energy}=\overset{M-1}{\underset{f_{1}=0}{\sum }}\overset{M-1}{\underset{f_{2}=0}{\sum }}\cdots \overset{M-1}{\underset{f_{n}=0}{\sum }}p{\left(f_{1},f_{2},\ldots ,f_{n}\right)}^{2}.$$

The energy, also called angular second moment, is the opposite of the entropy, measuring the order, the uniformity within the texture with respect to the considered local feature.

(B.4) $$\begin{array}{cc} & \text{Local homogeneity}\\ & \;=\overset{M-1}{\underset{f_{1}=0}{\sum }}\overset{M-1}{\underset{f_{2}=0}{\sum }}\cdots \overset{M-1}{\underset{f_{n}=0}{\sum }}\frac{p\left(f_{1},f_{2},\ldots ,f_{n}\right)}{1+\sum_{u=1}^{n-1}\sum_{v=u+1}^{n}\text{dif}{\left(f_{u},f_{v}\right)}^{2}}. \end{array}$$

The local homogeneity characterizes the texture from the point of view of the similarity of the pixels with respect to the considered feature, having increased values when the difference between the feature values of the corresponding pixels is decreased. This feature measures the closeness of the distribution of the elements in the GLCM to the GLCM diagonal.

The variance was computed as indicated;

(B.5) $$\text{Variance}=\sqrt{\sigma_{1}^{2}\sigma_{2}^{2}\cdots \sigma_{n}^{2}}.$$

The generalized variance, defined in (B.5), characterizes the texture from the point of view of the spreading of the considered feature values inside the cooccurrence matrix.

(B.6) $$\begin{array}{cc} & \text{Correlation}\\ & \;=\overset{N_{g}-1}{\underset{f_{1}=0}{\sum }}\;\overset{N_{g}-1}{\underset{f_{2}=0}{\sum }}\cdots \overset{N_{g}-1}{\underset{f_{n}=0}{\sum }}\left(\overset{n-1}{\underset{u=1}{\sum }}\;\overset{n}{\underset{v=u+1}{\sum }}\frac{\left(f_{u}-\mu_{u}\right)\left(f_{v}-\mu_{v}\right)}{\sqrt{\sigma_{u}^{2}\sigma_{v}^{2}}}\right)\\ & \;\;\times p\left(f_{1},f_{2},\ldots f_{n}\right). \end{array}$$

The *correlation, *mathematically defined in (B.6), expresses the linear dependence, between every two values of the local features, that are met inside the same *n*-tuple, respecting the spatial relation established by the displacement vectors. The mean and variance of the superior order cooccurrence matrices, with respect to the reference pixel (*μ*_1_, *σ*_1_), are described:

(B.7) $$\mu_{1}=\overset{M-1}{\underset{f_{1}=0}{\sum }}f_{1}\times \overset{M-1}{\underset{f_{2}=0}{\sum }}\cdots \overset{M-1}{\underset{f_{n}=0}{\sum }}p\left(f_{1},f_{2},\ldots ,f_{n}\right).$$

The mean of the superior order cooccurrence matrices with respect to the neighboring pixels (*σ*_*k*,_ *σ*_*n*,_) was computed according to the definitions described below:

(B.8) $$\begin{array}{cc} \mu_{k} & =\overset{M-1}{\underset{f_{k}=0}{\sum }}f_{k}\\ & \times \overset{M-1}{\underset{f_{1}=0}{\sum }}\cdots \overset{M-1}{\underset{f_{k-1}=0}{\sum }}\overset{M-1}{\underset{f_{k+1}=0}{\sum }}\cdots \overset{M-1}{\underset{f_{n}=0}{\sum }}p\left(f_{1},f_{2},\ldots ,f_{n}\right),\\ & \;\;\;\;\;\;\;\;\;\;\;\;\;\;\;\;\;\;\;k\geq 2,k<n,\\ & \mu_{n}=\overset{M-1}{\underset{f_{n}=0}{\sum }}f_{n}\times \overset{M-1}{\underset{f_{1}=0}{\sum }}\overset{M-1}{\underset{f_{2}=0}{\sum }}\cdots \overset{M-1}{\underset{f_{n-1}=0}{\sum }}p\left(f_{1},f_{2},\ldots ,f_{n}\right). \end{array}$$

The variance of the superior order cooccurrence matrices, with respect to the reference pixel (*σ*_1_), is described:

(B.9) $$\sigma_{1}^{2}=\overset{M-1}{\underset{f_{1}=0}{\sum }}{\left(f_{1}-\mu_{1}\right)}^{2}\times \overset{M-1}{\underset{f_{2}=0}{\sum }}\cdots \overset{M-1}{\underset{f_{n}=0}{\sum }}p\left(f_{1},f_{2},\ldots f_{n}\right).$$

The variance of the superior order cooccurrence matrices with respect to the neighboring pixels (*σ*_*k*,_ *σ*_*n*,_) was computed according to the definitions described below:

(B.10) $$\begin{array}{cc} \sigma_{k}^{2} & =\overset{M-1}{\underset{f_{k}=0}{\sum }}{\left(f_{k}-\mu_{k}\right)}^{2}\\ & \times \overset{M-1}{\underset{f_{1}=0}{\sum }}\cdots \overset{M-1}{\underset{f_{k-1}=0}{\sum }}\overset{M-1}{\underset{f_{k+1}=0}{\sum }}\cdots \overset{M-1}{\underset{f_{n}=0}{\sum }}p\left(f_{1},f_{2},\ldots ,f_{n}\right),\\ & \;\;\;\;\;\;\;\;\;\;\;\;\;\;\;\;\;\;\;k\geq 2,k<n,\\ \sigma_{n}^{2} & =\overset{M-1}{\underset{f_{n}=0}{\sum }}{\left(f_{n}-\mu_{n}\right)}^{2}\\ & \times \overset{M-1}{\underset{f_{1}=0}{\sum }}\overset{M-1}{\underset{f_{}=0}{\sum }}\cdots \overset{M-1}{\underset{f_{n-1}=0}{\sum }}p\left(f_{1},f_{2},\ldots ,f_{n}\right). \end{array}$$

The maximum probability, defined below, represents the maximum value that appears in the cooccurrence matrix and also highlights the pair, or *n*-tuple of feature values, that appears most frequently in the region of interest, the corresponding pixels respecting the spatial relation specified by the displacement vectors,

(B.11) $$p_{\max}=\mathrm{Max}\left(p\left(f_{1},f_{2},\ldots ,f_{n}\right),0<f_{k}<M,\;0\leq k\leq n\right).$$

### C. The Role of the Texture Analysis Methods in Emphasizing the Tumor Characteristics

In Figure 15, we can visualize the specific properties of the edges inside the tumoral tissue. Thus, the edge contrast and edge orientation variability are higher inside the malignant tumors while inside the benign tumors the edge contrast and density are more decreased. In Figure 16, the density of the spot microstructures inside the tumoral region is put into evidence. The spot frequency is higher inside the malignant tumors, emphasizing tissue regions severely affected by fibrosis, or containing an increased number of fatty cells. Inside the benign tumors, the spot microstructures are sparse.
